# *C*. *elegans miro-1* Mutation Reduces the Amount of Mitochondria and Extends Life Span

**DOI:** 10.1371/journal.pone.0153233

**Published:** 2016-04-11

**Authors:** Yanqing Shen, Li Fang Ng, Natarie Pei Wen Low, Thilo Hagen, Jan Gruber, Takao Inoue

**Affiliations:** 1 Department of Biochemistry, Yong Loo Lin School of Medicine, National University of Singapore, Singapore, Singapore; 2 Science Division, Yale-NUS College, Singapore, Singapore; Brown University/Harvard, UNITED STATES

## Abstract

Mitochondria play a critical role in aging, however, the underlying mechanism is not well understood. We found that a mutation disrupting the *C*. *elegans* homolog of Miro GTPase (*miro-1*) extends life span. This phenotype requires simultaneous loss of *miro-1* from multiple tissues including muscles and neurons, and is dependent on *daf-16/FOXO*. Notably, the amount of mitochondria in the *miro-1* mutant is reduced to approximately 50% of the wild-type. Despite this reduction, oxygen consumption is only weakly reduced, suggesting that mitochondria of *miro-1* mutants are more active than wild-type mitochondria. The ROS damage is slightly reduced and the mitochondrial unfolded protein response pathway is weakly activated in *miro-1* mutants. Unlike previously described long-lived mitochondrial electron transport chain mutants, *miro-1* mutants have normal growth rate. These results suggest that the reduction in the amount of mitochondria can affect the life span of an organism through activation of stress pathways.

## Introduction

Mitochondria are subcellular organelles of eukaryotes responsible for production of ATP through the aerobic metabolism as well as other key aspects of cellular metabolism and calcium homeostasis. In the inner membrane of mitochondria, the electron transport chain (ETC) couples the oxidation of NADH to establishment of a proton (H^+^) gradient across the membrane. This electrochemical gradient is used by the ATP synthase to drive synthesis of ATP. For over 50 years, one of the leading theories of aging, the free radical theory, sought the principal cause of aging in reactive oxygen species (ROS), which non-specifically react with and damage cellular components like protein and DNA [1]. Since it was recognized that production of ATP in mitochondria inevitably produces a small but significant quantity of ROS [2, 3], mitochondria were proposed as the main site of ROS production as well as the major target of ROS-induced damage [4]. Accumulation of ROS-induced damage throughout the life span of an organism was proposed to be a contributing cause of the age-dependent decline in cellular and organismal function and the decline in mitochondrial structure and function in particular.

In general, molecular genetic analyses of aging in model systems experimentally confirm the importance of mitochondria in aging. For example, in *C*. *elegans*, identification and characterization of long-lived mutants affecting ETC components [5, 6] as well as genome-wide screens for genes that affect the life span [7, 8] clearly indicate that disruption of mitochondrial ETC can and often does leads to the long-lived phenotype [9]. Evidence for a causal role of oxidative damage in lifespan determination of *C*. *elegans* is far less convincing [10]. Detailed analyses looking at mitochondrial ROS and oxidative damage show that the role of mitochondria in lifespan determination is complex. For example, simply attempting to increase or decrease mitochondrial ROS (e.g. by mutating the mitochondrial superoxide dismutase (SOD) gene) often did not lead to the predicted outcome (e.g. [11]). Also, measurements of ROS and ROS-induced damage in mutant animals failed to show a straightforward correlation between the ROS levels, oxidative damage and life span [11, 12]. Proposed explanations for these results include compensatory effects of protective feedback mechanism in response to low-level ROS (mitohormesis) [13] as well as gross changes to mitochondrial physiology [14, 15]. More recent studies further extend these ideas by proposing that effects of disrupting ETC on life span are mediated by specific stress signaling mechanisms. In particular, activation of the mitochondrial unfolded protein response pathway has been proposed to underlie lifespan extension in some ETC mutants [16, 17].

In addition to their metabolic activity, it is important to note that the structure and the amount of mitochondria are highly regulated. In cells, mitochondria undergo fusion and fission to switch between small disconnected structures (fragmented state) or large interconnected networks. This variation makes use of mitochondria number misleading when discussing the amount of mitochondria. Instead, the amount of mitochondria can be determined through various means, such as quantitation of mtDNA copy number, or microscope image analysis of fluorescently labeled mitochondria. Dedicated molecular systems exist to regulate mitochondrial fusion and fission as well as the amount of mitochondria [18, 19]. Mitochondrial fusion requires a set of proteins including Opa1 (*C*. *elegans eat-3*) and mitofusin (*C*. *elegans fzo-1*), whereas mitochondrial fission requires Drp1 (*C*. *elegans drp-1*). There is some evidence that mitochondrial fusion and fission may influence aging. Disrupting *drp-1* extends life span in specific genetic backgrounds [20]. On the other hand, the amount of mitochondria is regulated through control of mitochondrial biogenesis by factors including PGC-1α and AMPK [21]. In addition, unnecessary mitochondria are degraded through mitophagy (autophagy of mitochondria). Importantly, mitophagy can preferentially target damaged mitochondria for degradation. Thus, the turnover of mitochondria also serves as a quality control mechanism. Disruption of mitophagy also affects the life span of *C*. *elegans* [22–24].

Another factor affecting mitochondrial function is mitochondrial transport. The movement of mitochondria requires Miro (Mitochondrial Rho GTPase), an aberrant member of the small GTPase superfamily found in most eukaryotes [25, 26]. Miro has a unique domain architecture, consisting of two separate GTPase domains separated by a linker region containing Ca^++^ binding EF-hand motifs. A transmembrane region located at the C-terminus anchors the protein to the outer membrane of mitochondria, with GTPase domains and EF-hands located in the cytoplasm. Miro and its cytoplasmic binding partner Milton/TRAK link mitochondria to kinesin and dynein molecular motors in various cell types [27–30]. In neurons, Miro is responsible for localization of mitochondria to synapses, and mutations affecting Miro can lead to defective synaptic transmission [31, 32]. In axons, both anterograde and retrograde movement require Miro [33]. Aside from mitochondrial localization in neurons, additional functions are attributed to Miro. In *S*. *cerevisiae*, Miro (Gem1p) is responsible for proper segregation of mitochondria during cell division [28]. Interestingly, PINK1 and Parkin, components regulating clearance of damaged mitochondria, also regulate Miro, probably because arrest of mitochondrial movement is important for mitochondrial degradation through mitophagy [34]. Thus, Miro affects various aspects of mitochondrial biology, including subcellular localization, segregation during cell division and turnover.

Here, we report that a *C*. *elegans* Miro mutation extends the life span. This phenotype requires simultaneous loss of Miro from multiple tissues including neurons and muscles. The life span extension is dependent on activation of the *daf-16/FOXO* pathway, and reduction in synaptic transmission may contribute to this phenotype. In addition, we found that the Miro mutant contains about half the amount of mtDNA compared to the wild type, and by fluorescence microscopy, contain less mitochondria in body wall muscles and hypodermis. This does not appear to have any effect on growth rate or brood size. Oxygen consumption and ROS production are slightly reduced in the mutant, and the UPR^mt^ pathway is weakly activated and likely contributes to the life span extension. These characteristics, especially the absence of strong metabolic effects, set the Miro mutant apart from other long-lived *C*. *elegans* mutants affecting mitochondria such as *isp-1* and *clk-1* [5, 6, 9, 16]. We propose that this Miro mutation may extend the life span through a mechanism possibly related to the reduced amount of mitochondria.

## Results

### Loss of *miro-1* in neurons and muscles extends the life span of *C*. *elegans*

The *C*. *elegans* genome contains three genes related to Miro, *miro-1* (K08F11.5), *miro-2* (C47C12.4) and *miro-3* (Y47G6A.27). Sequences of these genes are highly similar at the nucleotide level (>90% identity). However, *miro-2* and *miro-3* contain deletions that remove conserved sequence features, and the genome of a closely related species, *Caenorhabditis briggsae* contains only the ortholog of *miro-1* in the syntenic position. Thus, *miro-2* and *miro-3* likely arose from recent gene duplication events and may not be functional.

**Fig 1:**
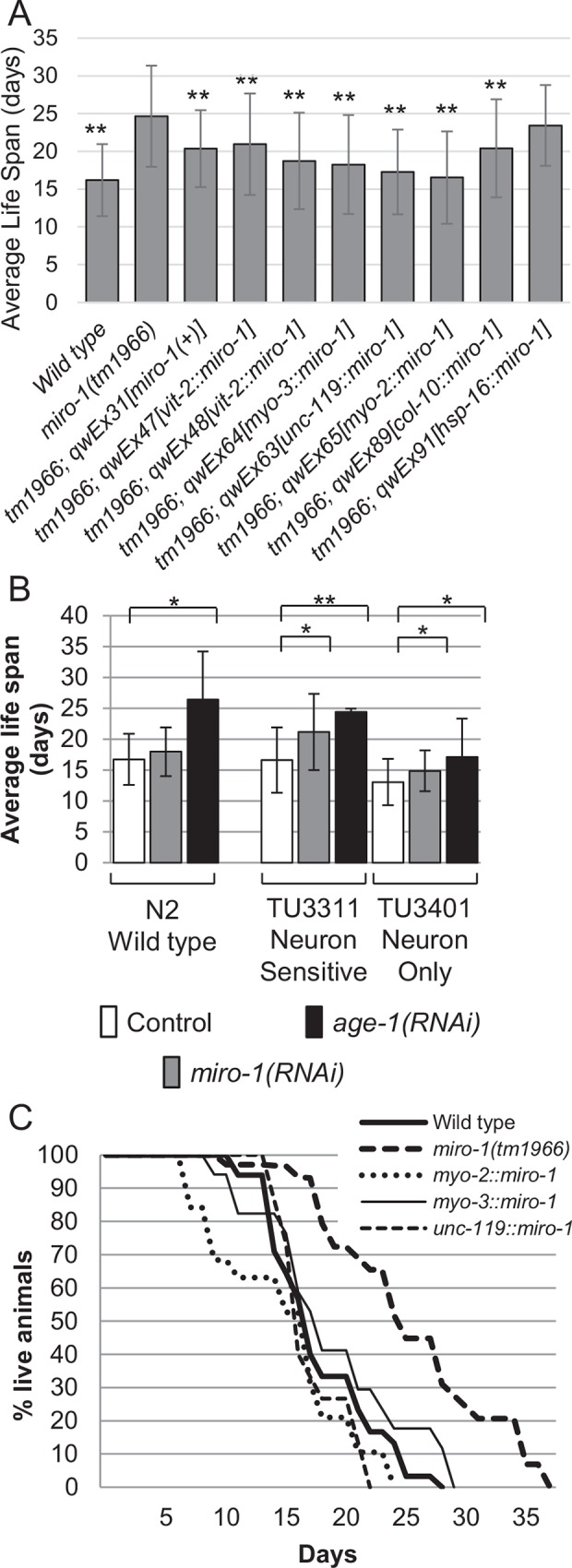
Extension of life span by the *miro-1(tm1966)* mutation. Average and standard deviation are shown. * p < 0.05, ** p < 0.0001 Student's t-test for comparison with *miro-1(tm1966)*. A. Life span of the wild type, *miro-1(tm1966)* and *miro-1(tm1966)* mutants carrying transgenes that express wild-type *miro-1* in specific tissues. These show combined data from multiple experiments. Results of individual experiments are in S1 Table. B. Life span of wild-type (N2) and RNAi-sensitized strains exposed to *miro-1(RNAi)*. C. Representative survival curve of *miro-1* mutants and rescued strains.

Because of the role of mitochondria in aging, we tested whether a *miro-1* mutation affects the life span of *C*. *elegans*. *miro-1(tm1966)* is a small deletion which removes parts of exon 4 and exon 5 and causes a frameshift leading to premature stop. In repeated experiments, we found that *miro-1(tm1966)* mutants lived significantly longer compared to the wild type (Fig 1A and 1C, S1 Table). Moreover, this phenotype was lost (rescued) when the wild-type *miro-1* gene was transgenically reintroduced into the *miro-1(tm1966)* strain, demonstrating that the long lived phenotype is caused by the loss of *miro-1* function.

To find out in which tissue *miro-1* functions to regulate life span, we carried out tissue-specific rescue experiments (Materials and Methods) (Fig 1A and 1C, S1 Table). We found that expression of wild-type *miro-1* in neurons under the control of the *unc-119* promoter, expression in body wall muscles under the control of the *myo-3* promoter, and expression in pharyngeal muscles under the control of the *myo-2* promoter all independently restored close to normal life span in the *miro-1(tm1966)* mutant background. Expression of *miro-1* in the intestine under the control of the *vit-2* promoter or expression in the hypodermis under the control of the *col-10* promoter weakly rescued the mutant phenotype. Finally, the *hsp-16*::*miro-1* construct, in which *miro-1* cDNA is placed downstream of the heat-shock inducible promoter did not extend the life span in the *miro-1(tm1966)* background in the absence of a heat-shock treatment, demonstrating that extrachromosomal arrays do not affect life span in the absence of *miro-1* expression and arguing against the possibility that *miro-1* coding sequence contains elements which can drive expression in *miro-1*-requiring tissue. Together, these results suggest that *miro-1* functions in multiple tissues to affect life span. Difference in the degree of rescue by different constructs may reflect distinct contribution of different tissues, or different expression levels from different constructs.

To further confirm that loss of *miro-1* extends the life span of *C*. *elegans*, we examined *miro-1* RNAi treated animals (Fig 1B). We found that the life span of wild-type *C*. *elegans* was extended by RNAi against *age-1/PI3K*, but not by RNAi against *miro-1*. This is consistent with neuronal function of *miro-1*, since neurons are not sensitive to RNAi in wild-type *C*. *elegans*. Therefore, we tested TU3311 *uIs60[unc-119*::*sid-1]*, a genetically engineered *C*. *elegans* strain with RNAi sensitive neurons [35]. (TU3311 expresses ectopically in neurons, *sid-1*, a gene required for RNAi sensitivity.) RNAi against *miro-1* significantly extended the life span of TU3311. Finally, we tested the TU3401 *sid-1; uIs69[unc-119*::*sid-1]* strain, which expresses *sid-1* only in neurons. RNAi against *miro-1* had a mild effect on the life span in the TU3401 mutant background. Based on these results, we conclude that reduction of *miro-1* in multiple tissues including neurons extend the life span of *C*. *elegans*.

### Extension of life span by *miro-1(tm1966)* requires *daf-16/FOXO*

In *C*. *elegans*, the insulin-related signaling pathway plays a major role in regulation of life span, with mutations in *daf-2/insulin receptor* and *age-1/PI3K* causing dramatic extensions of the life span [36, 37]. The effect of this pathway on aging is mediated by *daf-16*, encoding the FOXO transcription factor that activates a number of stress-resistance genes, including *sod-3* encoding a superoxide dismutase [38, 39].

**Fig 2:**
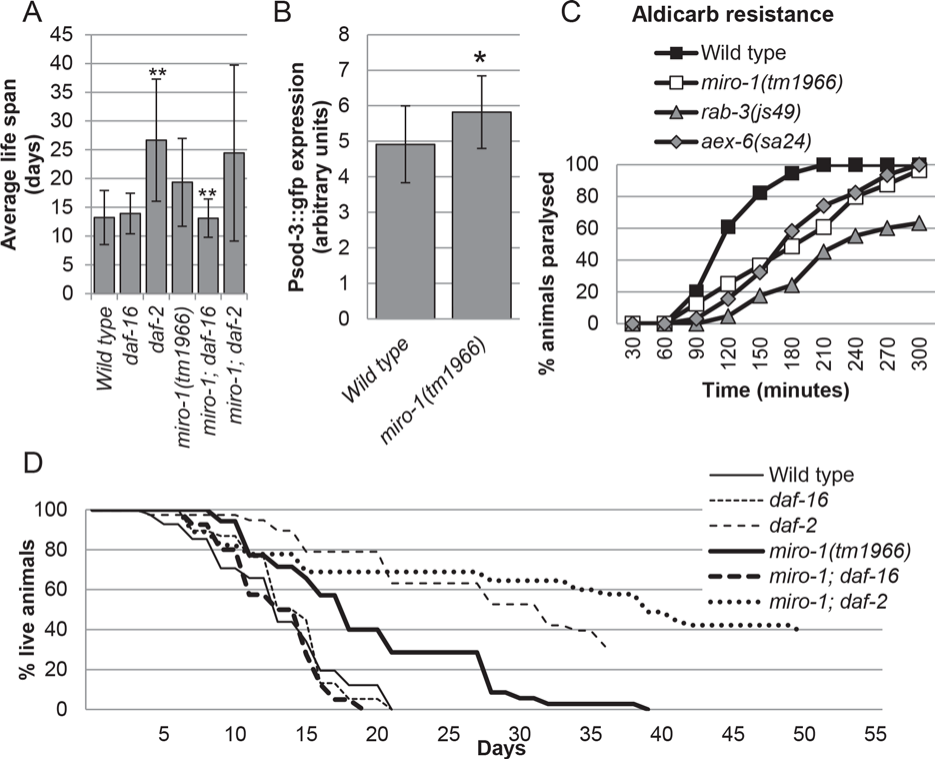
*daf-16/FOXO* dependent life span extension of *miro-1(tm1966)*. A. Life span of *miro-1* double mutants are shown (average +/- standard deviation). This assay was terminated after all *miro-1(tm1966)* animals had died, since *daf-2* mutants live very long time. The average life span was calculated for worms which died before the assay was terminated. Therefore, life spans for long lived *daf-2* and *miro-1; daf-2* double mutants are minimal possible values. B. Level of *sod-3*::*gfp* was quantified by taking fluorescence images and measuring mean brightness. C. Aldicarb resistance of *miro-1* and mutants with weak synaptic defects. D. Representative survival curve of double mutants.

We found that the *daf-16; miro-1* double mutants had a short life span similar to *daf-16* mutants, indicating that the life-span extension by *miro-1(tm1966)* requires *daf-16* (Fig 2A and 2D, S2 Table). To test whether the *daf-16* dependent stress resistance program is activated in the *miro-1* mutant, we examined the expression of *sod-3*::*gfp*, a GFP expression reporter for *sod-3* (Fig 2B). We found that GFP fluorescence from *sod-3*::*gfp* was increased significantly in the *miro-1(tm1966)* background compared to the wild-type background (Materials and Methods) (Fig 2B, S1 Fig). Thus, we conclude that *daf-16/FOXO* activity is likely increased in the *miro-1(tm1966)* mutant, contributing to the extended life span.

### *miro-1* mutants may have a mild defect in synaptic signaling

The contribution of neurons to the extension of life span suggested that the *miro-1* mutation may interfere with synaptic transmission. In *Drosophila* and in mammals, Miro proteins transport mitochondria to synaptic termini and are required for sustained synaptic transmission [31, 32]. In *C*. *elegans*, mutations that interfere with synaptic transmission extend life span through a *daf-16*/FOXO dependent mechanism [40] suggesting a possible role of neuronal *miro-1* in life span extension.

To determine whether synaptic transmission is affected, we tested the sensitivity of *miro-1(tm1966)* mutants to aldicarb, a cholinesterase inhibitor. Mutations affecting synaptic transmission confer varying degrees of aldicarb resistance [41]. Previously, RNAi targeting *miro-1* was found to cause mild resistance to aldicarb [42]. Consistently, we found that *miro-1(tm1966)* mutants had a slightly reduced sensitivity to aldicarb, similar to *aex-6(sa24)* mutants but weaker than *rab-3(js49)* mutants (Fig 2C). However, *aex-6(sa24)* and *rab-3(js49)* mutants are not long-lived to the same extent as *miro-1(tm1966)* (S3 Table). Thus, although synaptic transmission may be partially compromised, it is unlikely that this is the sole reason for the extension of life span in *miro-1*. More likely, partially compromised synaptic transmission acts synergistically with the effect of *miro-1* loss in muscles.

### *miro-1(tm1966)* affects the amount of mitochondria

**Fig 3:**
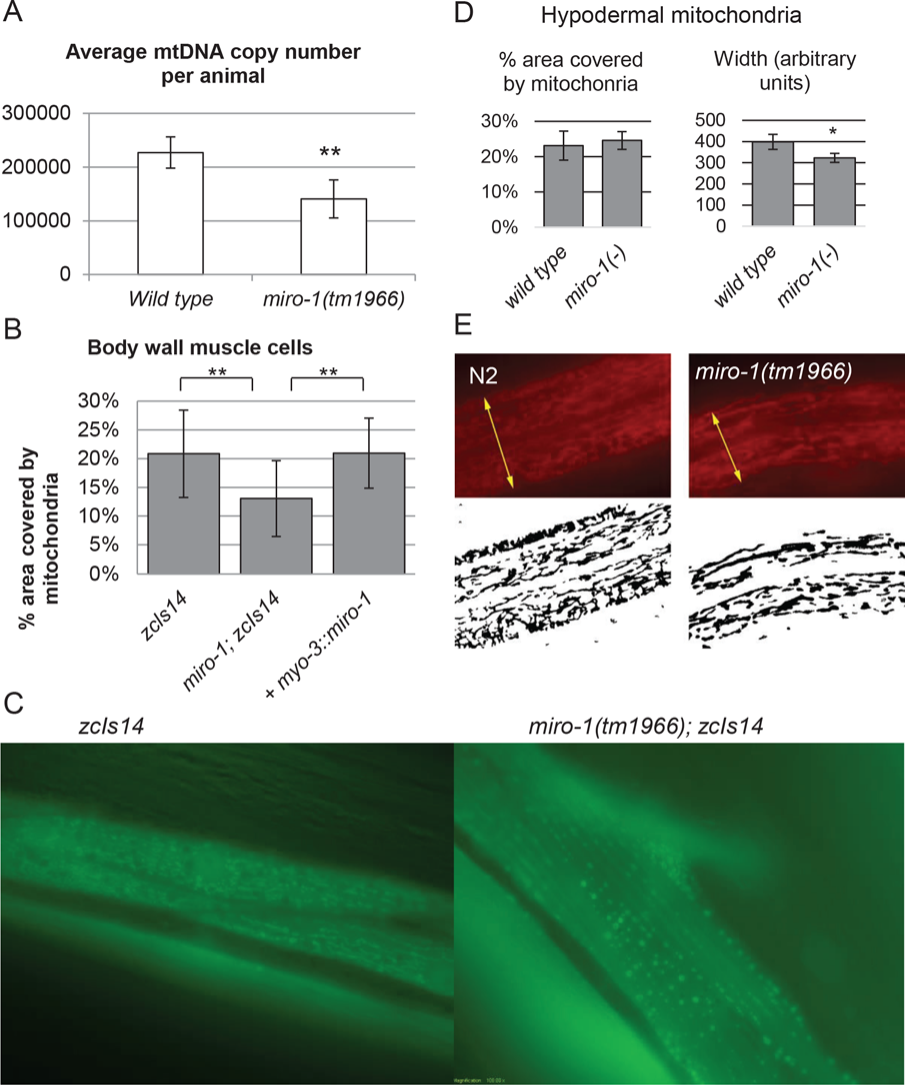
Reduced amount of mitochondria of *miro-1(tm1966)* mutants. A. qPCR was performed on RNA extracted from individual L3 animals to determine the mtDNA copy number. Average and standard deviation from 20 animals are shown. B. Percentage of muscle area covered by mitochondria, calculated from images similar to those shown in panel C. C. Fluorescence images of body wall muscle mitochondria in animal carrying *zcIs14[myo-3*::*mtGFP]*. Rhomboid shapes are individual muscle cells, and bright spots within are mitochondria. D. Hypodermal mitochondria. From images similar to those in panel E, the width of the band containing mitochondria (yellow arrows in E), and the density of mitochondria within this band were measured. Six wild-type and nine mutant animals were assayed. E. Hypodermal mitochondria stained with MitoTracker Red CMXRos. Original fluorescence images and thresholded image representing mitochondria are shown.

To determine the amount of mitochondria in *miro-1(tm1966)* mutants, we first quantified in single-worm qPCR measurements, the copy number of mitochondrial DNA (mtDNA). We found that *miro-1(tm1966)* mutants contained, on average, about half the number of mtDNA compared to the wild-type (Fig 3A).

To further test whether this reduction in mtDNA correlated with a reduction in the amount of mitochondria as observed under a microscope, we visualized hypodermal mitochondria using MitoTracker and body wall muscle mitochondria using mitochondria-targeted GFP (Materials and Methods) [43]. In *C*. *elegans* body wall muscle cells, mitochondria are concentrated in a single layer located below the muscle fibers. Using image analysis, we found that mitochondria covered approximately 20% of this layer in the wild-type body wall muscle (Fig 3B and 3C) (Materials and Methods). In contrast, in the *miro-1* mutant, the area covered was significantly reduced to approximately 12% (p<0.0001, Student's t-Test). This phenotype could be rescued by the reintroduction of the wild-type *miro-1* in the body wall muscle cells using the *myo-3*::*miro-1* construct. In the hypodermis, the amount of mitochondria also appeared to be reduced (Fig 3D and 3E). Specifically, hypodermal mitochondria are organized into bands of fixed width running along the length of the worm. In *miro-1(tm1966)* mutants, the width of the mitochondria-containing region was significantly reduced (p = 0.0017, Student's t-test), while the density was not affected, suggesting that the overall amount of hypodermal mitochondria is reduced.

### *miro-1(tm1966)* does not strongly affect oxygen consumption or ROS damage

Some life span extending mutations affecting the mitochondrial electron transport chain cause dramatic changes to the animal's oxygen metabolism. To test whether the *miro-1(tm1966)* mutation also alters the metabolism, we measured the oxygen consumption and ROS damage.

**Fig 4:**
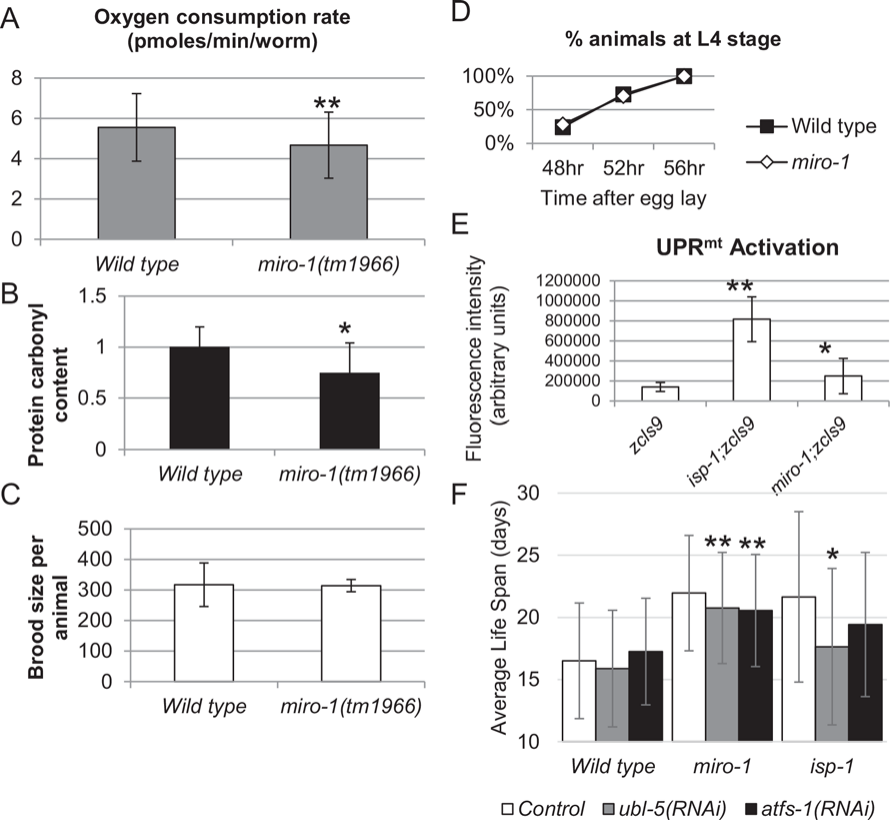
Effect of *miro-1* on mitochondrial function. A. Oxygen consumption was measured using the Seahorse XF analyzer. Average and standard deviation from three independent sets of assays at approximately 24°C are shown. Results obtained using a Clark electrode instrument showed no difference between the mutant and the wild type (Materials and Methods). B. Protein carbonyl content (product of oxidative damage to proteins). C. Brood size of individual animal. D. Growth rate of the wild-type and the *miro-1* mutant. Synchronized populations of wild-type and mutant eggs were allowed to grow for set lengths of time. Both *miro-1* and the wild type reach the L4 stage at the same time. E. Activation of UPR^mt^ was determined by examining fluorescence in *zcIs9[hsp-60*::*gfp]* reporter carrying strains, which is activated in response to UPR^mt^. * p < 0.05, ** p < 0.0001 Student's t-test. F. Life span of wild type, *miro-1(tm1966)* and *isp-1* mutant under RNAi treatment for *ubl-5* and *atfs-1*. Note, *isp-1* results are less significant because of a smaller number of animals which were assayed.

The oxygen consumption of *miro-1(tm1966)* and the wild type was measured using both a Clark electrode instrument and a Seahorse XF Analyzer (Materials and Methods). In repeated assays, we found that *miro-1(tm1966)* mutants had a slightly reduced oxygen consumption compared to the wild-type (Fig 4A), or were indistinguishable from the wild type (Materials and Methods). In most assays, the difference was not as dramatic as the difference in the mtDNA copy number (Fig 3A). Thus, reduction in mitochondrial mass probably does not lead to corresponding reduction in oxygen consumption by the whole animal. Rather, the amount of oxidative phosphorylation per worm likely depends on the need for ATP and therefore remains relatively constant despite reduction in the amount of mitochondria.

The damage caused by mitochondria-produced ROS was measured using the amount of protein carbonyl as a proxy (Materials and Methods). Defects in mitochondria can increase or decrease ROS production, depending on which mitochondrial process is disrupted. The amount of ROS damage found in the *miro-1(tm1966)* mutant was very slightly reduced compared to the wild-type. Together, these data show that *miro-1(tm1966)* maintain near normal oxygen consumption and experience slightly lower levels of oxidative damage under normal growth conditions, despite significantly decreased amount of mitochondria.

### The effect of *miro-1(tm1966)* on growth rate and brood size

Although a number of life span extending mitochondrial electron transport chain mutations are known, these mutations have a strong effect on the growth rate or the brood size [9]. We found that the growth rate was not strongly affected in the *miro-1(tm1966)* mutant (Fig 4D). The brood size was not obviously affected when the number of progeny from individual worms were counted. However, we did observe a low frequency of sterility in the *miro-1(tm1966)* mutant strain (Materials and Methods). Although gonadal defects can extend life span, because sterility is observed in less than 10% of *miro-1(tm1966)* animals, this cannot account for the long-lived phenotype of *miro-1(tm1966)*. Together with the absence of strong effect on oxygen consumption and ROS damage, we conclude that *miro-1(tm1966)* does not extend life by strongly disrupting the oxidative phosphorylation metabolism.

### The effect of *miro-1(tm1966)* on the UPR^mt^ pathway

Activation of mitochondrial unfolded protein response pathway (UPR^mt^) was suggested to underlie life span extension in many long-lived ETC mutations [16]. To determine whether *miro-1(tm1966)* extends the life span through this mechanism, we examined the expression of an expression reporter *zcIs9[hsp-60*::*gfp]* [43]. *hsp-60* is a mitochondrial heat shock protein whose transcription is increased when UPR^mt^ is activated. We found that in the *miro-1(tm1966)* mutant background, the expression of *zcIs9* reporter was weakly but significantly increased compared to the wild-type background (p = 0.0009, Student's t-test) (Fig 4E).

UPR^mt^ activation requires a number of dedicated factors including *ubl-5* (ubiquitin like protein) and *atfs-1* (activating transcription factor associated with stress). However, a recent report indicates that *ubl-5*, but not *atfs-1* is required for the long-lived phenotype of ETC mutants, raising doubt as to whether UPR^mt^ is really responsible for the phenotype [44]. To test whether the activation of UPR^mt^ or *ubl-5* contributes to the extended life span of *miro-1* mutants, we disrupted *ubl-5* and *atfs-1* using RNAi (Fig 4F, S4 Table). We found that *ubl-5(RNAi)* weakly suppressed the long-lived phenotype of *miro-1* mutant but had not obvious effect on the life span of the wild type. Surprisingly, we also found that *atfs-1(RNAi)* also weakly suppressed the long-lived phenotype of *miro-1* without affecting the wild type. These results suggest that activation of UPR^mt^ or a related pathway contributes to the long-lived phenotype of the *miro-1* mutant.

## Discussion

### Loss of *miro-1* extends life span through multiple mechanisms

**Fig 5:**
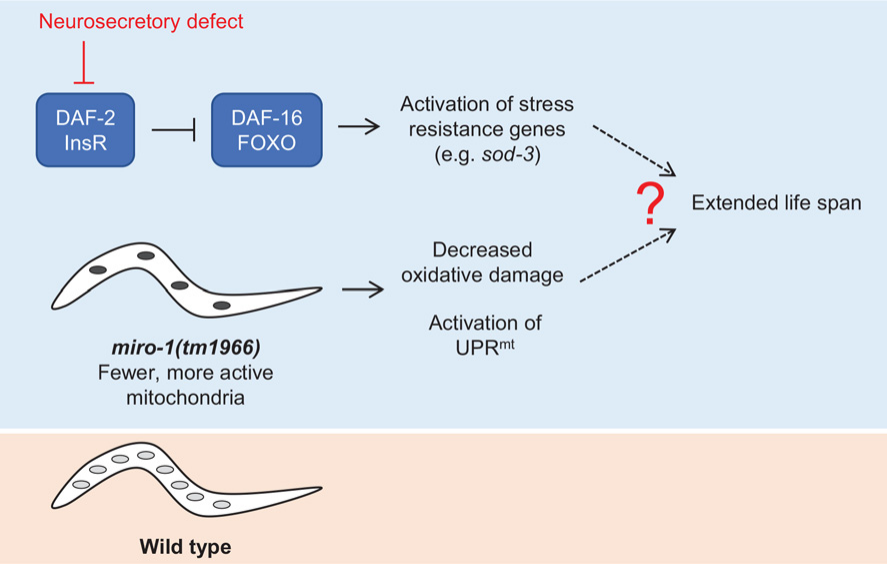
Possible mechanism of life span extension by the *miro-1(tm1966)* mutation. On one hand (upper branch), the defect in *miro-1* may cause a neurosecretory defect which activates the *daf-16*-dependent stress resistance pathway. At the same time *miro-1(tm1966)* contains less mitochondria than the wild type (bottom branch). This may contribute to the phenotype through reduced ROS production or activation of a mitochondria stress resistance pathway.

We found that a mutation affecting the *C*. *elegans* mitochondrial GTPase gene *miro-1* extends life span. This phenotype is dependent on loss of *miro-1* function in multiple tissues including neurons, with loss in multiple tissues contributing to the phenotype. Extension of life span by *miro-1(RNAi)* on *C*. *elegans* strain with engineered neuronal RNAi sensitivity, but not wild-type *C*. *elegans*, confirms that this phenotype is caused by loss of *miro-1* and that loss in multiple tissues including neurons is required for life span extension. In other words, the increased life span of the *miro-1(tm1966)* arises from synergistic effects of this mutation in multiple tissues (Fig 5).

At least one effect of *miro-1* disruption is the activation of the *daf-16/FOXO* stress resistance program. This is evident from the fact that the life span extension of *miro-1(tm1966)* is suppressed by a *daf-16* mutation, and the fact that *sod-3*, a downstream target of *daf-16*, is weakly upregulated in the *miro-1* mutant. Given the known importance of Miro proteins to sustained neuronal transmission in *Drosophila* and mice [31, 32], it is possible that loss of Miro leads to a neurosecretory defect, which causes extension of life span by a *daf-16/FOXO* dependent mechanism [40]. Weak aldicarb resistance observed in *miro-1(tm1966)* is consistent with this idea. However, it is important to note that this cannot be the sole cause of life span extension, since loss of *miro-1* from multiple tissues is required for this phenotype.

### Effect of reduced mitochondrial mass on metabolism and life span

Another possible contributor to the life span extension is the dramatic reduction in the amount of mitochondria. This reduction is indicated by the number of mtDNA, as well through microscopic observation of mitochondria in body wall muscles and the hypodermis. Interestingly, we did not find evidence for a strong effect of this reduction on metabolism including oxygen consumption or ROS damage. During ATP synthesis, electrons are transferred along a series of electron carriers that form the mitochondrial electron transfer chain (ETC). When these electron carriers are in a reduced state and molecular oxygen is present, electrons can also tunnel to oxygen, resulting in one-electron reduction of oxygen and production of superoxide (O_2_*^-^). In isolated mitochondria, several ETC sites and mechanisms have been identified that lead to the production of O2*^-^ in the ETC [45]. Mitochondria that are actively generating ATP from ADP ("state 3") produce negligible amounts of ROS. However, when ATP synthesis rate is low because the amount of ADP is limiting ("state 4"), the electron carriers in the ETC become highly reduced, promoting the tunneling of electrons to oxygen, resulting in significant ROS production. Thus if *miro-1* mutants are maintaining high respiration rate despite reduction in ETC in the amount of components, this may lead to reduced ROS production.

On the other hand, it is questionable whether the mild reduction in ROS damage we observed can account for the strong extension of life span in *miro-1(tm1966)*. The relationship between ROS and life span is complex, and oxidative damage does not correlate well with life span in many mutants [12]. Moreover, reduced amount of mtDNA and reduced volume of mitochondria (as observed using fluorescence imaging) may not correlate with the amount of respiratory capacity which is present.

Perhaps a more likely possibility is that reduced mitochondrial mass may induce mitochondrial stress signals that lead to life span extension. We observed a slight activation of UPR^mt^ in *miro-1(tm1966)*, which was weaker than the level of activation observed in a long-lived ETC mutant, *isp-1*. Additionally, disruption of *ubl-5* or *atfs-1* by RNAi weakly suppressed the long-lived phenotype. These results suggest that weak activation of a mitochondrial stress program may act synergistically with activation of *daf-16/FOXO* to extend the life span.

It is also important to note that mitochondria have a number of functions other than ATP production through oxidative phosphorylation. Disruption of these functions may also contribute to the phenotype of the *miro-1* mutant.

### Miro and regulation of mitochondrial turnover

Finally, it is also possible that the life span extension by *miro-1* loss is related to the role of Miro in mitochondrial turnover, which also has an effect on life span [24]. As a target of Parkin/PINK1-mediated mitochondrial quality control system, mammalian Miro negatively regulates mitochondrial turnover by mitophagy. If this mechanism is conserved in *C*. *elegans*, loss of *miro-1* may increase the turnover rate of mitochondria. On one hand this may be the underlying cause of reduction in mitochondrial mass we observed. On the other hand, this change to mitochondrial turnover may affect aging more directly by improving the quality control of mitochondria in *miro-1* mutants, thereby causing the mutants to live longer.

## Materials and Methods

### K08F11.5 encodes *C*. *elegans* Miro (mitochondrial Rho)

BLAST searches of predicted *C*. *elegans* proteins revealed three potential members of the Miro protein family in *C*. *elegans*: K08F11.5 (*miro-1*), C47C12.4 (*miro-2*) and Y47G6A.27 (*miro-3*). Alignment of genome and predicted protein sequences of these genes revealed that K08F11.5 encoded a protein with homology to mammalian Miro along its entire length. However, C47C12.4 and Y47G6A.27 had nucleotide sequences very similar to K08F11.5 but with deletions which removed some functional domains of Miro. Thus, C47C12.4 and Y47G6A.27 are likely to be results of recent gene duplications with partial degradation of the sequence following the duplication event.

### RT-PCR analysis of mtDNA copy number

MtDNA copy number was quantified as previously described in [46, 47]. Briefly, individual worms were picked into a PCR tube containing 50 μl of worm lysis buffer, lysed and mtDNA copy number determined by quantitative real-time PCR (qRT-PCR). The assay was performed in parallel with a reference sample of a known copy number (serial dilutions of a previously quantified worm lysate).

### Protein carbonyl determination

Oxidative damages to proteins generate amino acid side chains with carbonyl groups. To determine the level of oxidative damage to proteins, the level of protein carbonyls was determined as described [48]. Briefly, worms were transferred into micro-centrifuges tubes and washed in M9 buffer to remove bacteria and debris. Worms were then resuspended in 100 μl of PBS-T lysis buffer (0.1% Tween-20 in phosphate-buffered saline solution containing 1 mM phenylmethylsulfonyl fluoride protease inhibitor (PMSF) and 50 mM of dithiothreitol (DTT)) and sonicated on ice. Next, carbonyl groups in the sample were derivatised with 2,4-dinitrophenylhydrazine according to the manufacturer's protocol (OxyBlot Protein Oxidation Detection Kit). The lysates were then transferred to a nitrocellulose membrane using a slot blot apparatus, and probed with anti-2,4-dinitrophenylhydrazine primary antibody, followed by HRP conjugated secondary antibody. Finally, ECL reaction was carried out and the level of signal was quantified. The assay was repeated three times, with eight measurements per strain in a typical assay.

### Observation of mitochondria

Hypodermal mitochondria were labeled by staining with MitoTracker Red CMXRos (Invitrogen) and observed using Olympus BX51 microscope equipped for Nomarski and fluorescence microscopy. Young adult worms were either stained in MitoTracker solution for 20 minutes or fed with MitoTracker treated OP50 for 30 minutes. After staining, worms were destained for 20 minutes. Stained worms were mounted as described [49] and observed using Olympus BX51 microscope equipped for Nomarski and fluorescence microscopy. The width of the mitochondria containing region was determined using the Olympus cellSens, ImageJ or Photoshop (Adobe).

Body wall muscle mitochondria were labeled by mitochondria-targeted green fluorescent protein (mtGFP) expressed in the body wall muscle (*zcIs14)* [43]. Using the ImageJ software, from a fluorescence image, an outline of a single muscle cell was selected. The area covered by mitochondria was determined by thresholding the pixel intensity, and the percentage of area covered by mitochondria (as a fraction of the total muscle area) was calculated.

### Aldicarb assay

The aldicarb assay was done as described in [41]. 30–40 adult worms were placed on NGM plates supplemented with 1 mM aldicarb, and the number of paralyzed worms was determined every 30 minutes.

### Life span assay

L4 worms were placed on seeded NGM plates and transferred to fresh plates every 2 days until all worms stopped laying eggs. The number of worms that died of old age (no obvious injury) was calculated excluding all worms that were missing, exploded, bagged or desiccated.

### *Caenorhabditis elegans* strains

*miro-1(tm1966)* mutants were obtained from the Mitani laboratory. The mutation was outcrossed twice to obtain the strain ZF1027 which was the standard *miro-1(tm1966)* strain used in this study. *C*. *elegans* strains were maintained as described [50]. Mutations and transgenes used in this study include: *miro-1(tm1966)*, *aex-6(sa24)*, *rab-3(js49)*, *daf-2(e1370)*, *daf-16(mgDf50)*, *sid-1(pk3321)*, *zcls14[myo-3*::*mtGFP]*, *zcIs9[hsp-60*::*gfp]*, *muIs84[sod-3*::*gfp]*, *qwEx31[miro-1(+)]*, *qwEx47[vit-2(6)*::*miro-1(+)]*, *qwEx48[vit-2(9)*::*miro-1(+)]*, *qwEx64[myo-3*::*miro-1(+)]*, *qwEx63[unc-119*::*miro-1(+)]*, *qwEx65[myo-2*::*miro-1*(+)], *qwEx91[hsp-16*::*miro-1(+)]* and *qwEx89[col-10*::*miro-1(+)]*, *uIs60[unc-119*::*YFP*, *unc-119*::*sid-1]*, *uIs69[myo-2*::*mCherry*, *unc-119*::*sid-1]*.

### Transgenic strains and RNAi

For the rescue of *miro-1(tm1966)*, a genomic fragment containing the entire *miro-1* gene was amplified from genomic DNA by PCR using primers cgcgGGTACCgaatgagcgacgacgagacgtt and cgcgACTAGTagttccggatagtaacaaatcct. Resulting 4.5 kb fragment was purified and injected into *miro-1* mutants along with *myo-2*::*yfp* (L4640) coinjection marker. RNAi was induced by feeding dsRNA expressing *E*. *coli* to worms [51]. With the exception of *miro-1(RNAi)*, RNAi feeding bacteria were obtained from the RNAi library [52]. To construct the RNAi feeding bacteria strain targeting *miro-1*, we cloned a 1.8kb genomic fragment into the L4440 vector. The resulting plasmid was transformed into HT115.

To express *miro-1* in different tissues, we first amplified *miro-1* cDNA from total RNA using primers cgcgGGTACCgaatgagcgacgacgagacgtt and cgcgACTAGTagttccggatagtaacaaatcct. The cDNA was sequence verified and cloned into vectors containing tissue-specific promoters, pPD157.63 (*vit-2*), L4816 *(myo-3*) and pPD191.45 *(col-10)* using restriction sites KpnI, and SpeI or PspOMI. For neuronal expression, the *unc-119* promoter was cut from the *unc-119* genomic DNA fragment [53] using HindIII and Sau3AI and cloned into pPD95.75 vector backbone before inserting *miro-1* cDNA. Similarly, for *hsp-16*, the promoter (derived from pPD49.78) and cDNA were cloned into the pPD95.77 vector.

### Measurement of brood size and growth rate

To measure brood size, individual L4 worms were placed on seeded NGM plates and allowed to lay eggs. Each worm was moved to a new plate every night until egg laying stopped and the number of progeny on each plate was counted and added to give the brood size per individual. To determine the frequency of sterile *miro-1(tm1966)* animals, 72 L4 larvae were picked to individual NGM plates. Two days later, 62 worms had progeny, five worms were sterile and five worms were found on the side of plates.

The growth rate was determined as the number of hours it took to reach adulthood. Ten adult worms with eggs were placed on a seeded NGM plate and allowed to lay eggs for four hours after which parents were removed. At fixed time points, the number of progeny that reached the adult stage was counted.

### Image analysis

To measure the level of *muIs84[sod-3*::*gfp]* and *zcIs9[hsp-60*::*gfp]* expression, fluorescence images of L4 and young adult worms were taken and analyzed using ImageJ. Briefly, the whole worm was selected and the mean grey values was determined.

### Oxygen Consumption Assay

Clark electrode assays were carried out with synchronized populations of late L4 worms. The worms were washed three times and resuspended in M9 buffer. The change in oxygen concentration was recorded over a period of 15 minutes, and repeated after reoxygenation. The M9 buffer in which the worms were suspended was collected, and re-assayed after removal of worms to control for presence of bacteria. The rate of oxygen consumption was then normalized against the total soluble protein in each sample after the sample was sonicated. The experiment was repeated twice, and neither showed significant difference between wild-type and *miro-1(tm1966)* mutants.

Seahorse XFe24 assays were carried out using synchronized populations placed in M9. Approximately 100 to 250 L4 worms were placed in each well. The experiment was repeated 6 times, with multiple wells for each genotype in each experiment. After the assay, the number of worms was counted and used to calculate the oxygen consumption rate per worm.

## Supporting Information

S1 Fig *sod-3*::*gfp* expression in the wild type, *miro-1* mutants and *daf-2* mutants. The expression level is *miro-1* is significantly higher than in the wild type but not as high as in *daf-2*.
(PDF)

S1 Table Life span of *miro-1* and rescue by tissue specific expression. (PDF)

S2 Table Life span of *daf-16* and *daf-2* double mutants. (PDF)

S3 Table Life span of *aex-6* and *rab-3*. (PDF)

S4 Table Life span of worms treated with *ubl-5* and *atfs-1* RNAi. (PDF)
