# Supplementary material for: C. elegans miro-1 Mutation Reduces the Amount of Mitochondria and Extends Life Span
Source: PLoS One. 2016 Apr 11;11(4):e0153233. doi: 10.1371/journal.pone.0153233 (PMC4827821; doi:10.1371/journal.pone.0153233)
Supplement: S1 Fig — The expression level is miro-1 is significantly higher than in the wild type but not as high as in daf-2. (PDF) [file pone.0153233.s001.pdf]

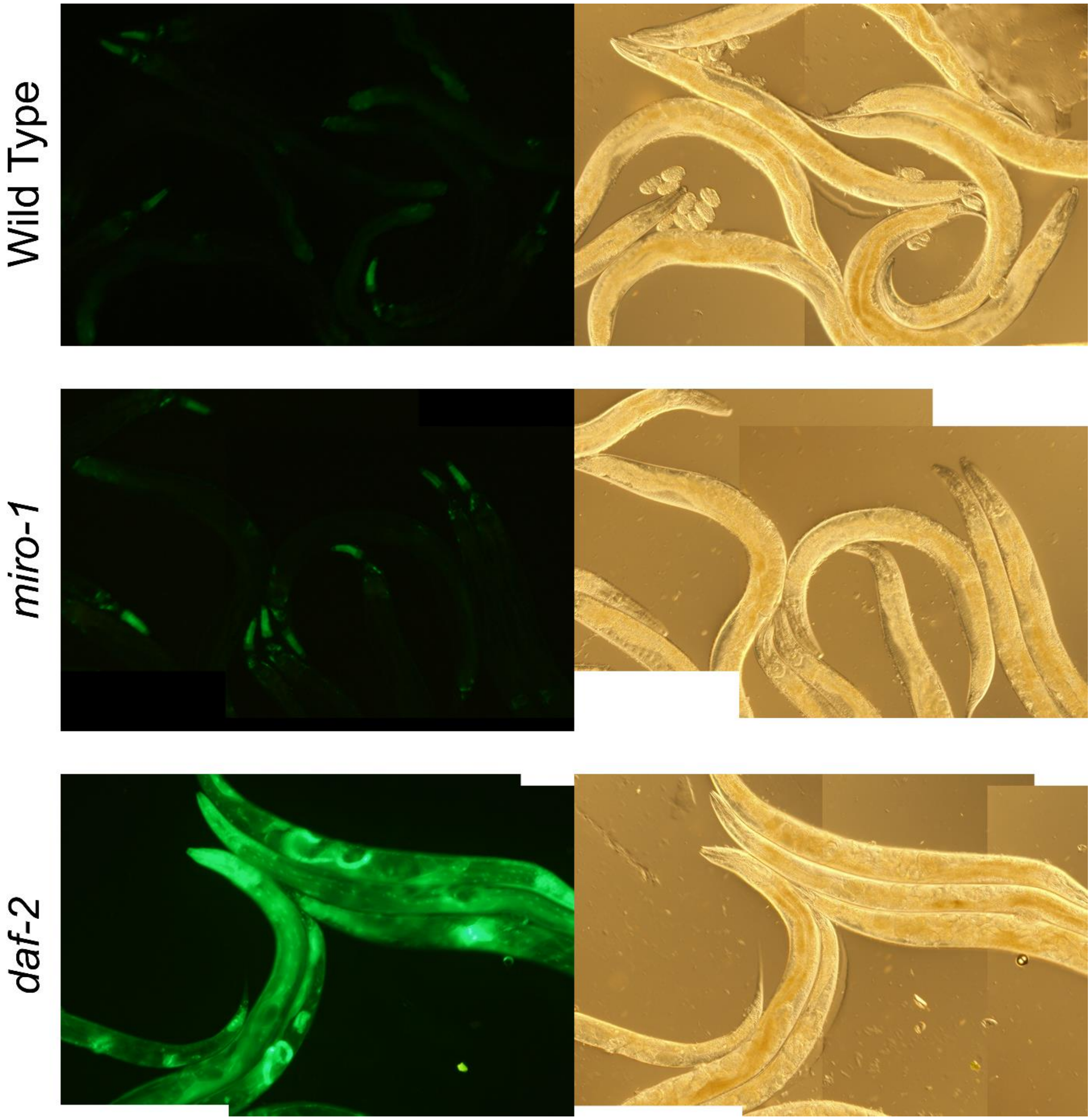

Supplemental Figure S1: *sod-3::gfp* expression in the wild type, *miro-1* mutants and *daf-2* mutants. The expression level is *miro-1* is significantly higher than in the wild type but not as high as in *daf-2*.
