# Supplementary material for: C. elegans miro-1 Mutation Reduces the Amount of Mitochondria and Extends Life Span
Source: PLoS One. 2016 Apr 11;11(4):e0153233. doi: 10.1371/journal.pone.0153233 (PMC4827821; doi:10.1371/journal.pone.0153233)
Supplement: S1 Table — (PDF) [file pone.0153233.s002.pdf]

Table S1: Life span of *miro-1* and rescue by tissue specific expression.

Experiment	Genotype	Lifespan (mean±S.D.)	n	p value vs wild type	p value vs miro-1(-)
1	Wild type	12.28±4.58	54		
	miro-1(tm1966)	21.91±7.52	47	<0.0001	
	miro-1(tm1966);qwEx31[miro-1(+)]	16.73±5.38	51	<0.0001	<0.0001
2	Wild type	17.15±6.17	59		
	miro-1(tm1966)	24.96±6.66	49	<0.0001	
	miro-1(tm1966);qwEx31[miro-1(+)]	18.46±5.05	46	0.25	<0.0001
3	Wild type	18.49±4.86	78		
	miro-1(tm1966)	25.06±5.79	68	<0.0001	
	miro-1(tm1966);qwEx47[vit-2(6)::miro-1(+)]	21.14±6.83	64	0.0087	0.0004
	miro-1(tm1966);qwEx48[vit-2(9)::miro-1(+)]	19.09±5.64	66	0.4924	<0.0001
4	Wild type	16.86±3.94	59		
	miro-1(tm1966)	25.29±5.79	41	<0.0001	
	miro-1(tm1966);qwEx31[miro-1(+)]	19.94±5.12	53	0.0005	<0.0001
	miro-1(tm1966);qwEx48[vit-2(9)::miro-1(+)]	19.56±6.80	41	0.0138	<0.0001
5	Wild type	15.35±3.30	55		
	miro-1(tm1966)	25.19±5.44	54	<0.0001	
	miro-1(tm1966);qwEx31[miro-1(+)]	20.78±5.08	50	<0.0001	<0.0001
	miro-1(tm1966);qwEx48[vit-2(9)::miro-1(+)]	18.33±5.99	55	0.0016	<0.0001
6	Wild type	15.53±4.26	30		
	miro-1(tm1966)	20.43±5.63	23	0.0007	
	miro-1(tm1966);qwEx47[vit-2(6)::miro-1(+)]	17.52±6.31	23	0.1415	0.106
	miro-1(tm1966);qwEx48[vit-2(9)::miro-1(+)]	17.61±7.38	21	0.209	0.1594
	miro-1(tm1966);qwEx64[myo-3::miro-1(+)]	13.62±6.3	23	0.1942	0.0004
	miro-1(tm1966);qwEx63[unc-119::miro-1(+)]	13.38±5.11	16	0.1355	0.0003
7	Wild type	17.9±4.48	30		
	miro-1(tm1966)	25.55±7.06	29	<0.0001	
	miro-1(tm1966)qwEx47[vit-2(6)::miro-1(+)]	24.14±5.08	21	<0.0001	0.4394
	miro-1(tm1966);qwEx48[vit-2(9)::miro-1(+)]	18.33±7.69	24	0.7983	0.0008
	miro-1(tm1966);qwEx64[myo-3::miro-1(+)]	18.94±6.16	17	0.5085	0.0025
	miro-1(tm1966);qwEx65[myo-2::miro-1(+)]	14.89±5.69	19	0.0447	0.001
	miro-1(tm1966);qwEx63[unc-119::miro-1(+)]	17.27±2.84	15	0.6227	<0.0001
8	Wild type	12.49±3.4	43		
	miro-1(tm1966)	22.23±6.05	39	<0.0001	
	miro-1(tm1966);qwEx64[myo-3::miro-1(+)]	18.36±5.54	36	<0.0001	0.0052
	miro-1(tm1966);qwEx65[myo-2::miro-1(+)]	16.3±5.76	44	0.0003	<0.0001
	miro-1(tm1966);qwEx63[unc-119::miro-1(+)]	16.98±6.69	42	0.0002	0.0004
	miro-1(tm1966);qwEx89[col-10::miro-1(+)]	19.89±4.79	38	<0.0001	0.0642
9	Wild type	15.37±5.22	43		
	miro-1(tm1966)	27.2±8.14	44	<0.0001	
	miro-1(tm1966);qwEx64[myo-3::miro-1(+)]	20.9±6.64	31	<0.0001	0.0002
	miro-1(tm1966);qwEx65[myo-2::miro-1(+)]	17.59±6.61	41	0.0906	<0.0001
	miro-1(tm1966);qwEx63[unc-119::miro-1(+)]	17.87±3.95	31	0.028	<0.0001
	miro-1(tm1966);qwEx89[col-10::miro-1(+)]	20.82±7.67	45	0.0002	0.0003
10	Wild type	16.93±5.61	41		
	miro-1(tm1966)	23.92±8.19	37	<0.0001	
	miro-1(tm1966);qwEx91[hsp-16::miro-1(+)]	23.43±5.36	40	<0.0001	0.7552
	miro-1(tm1966);qwEx63[unc-119::miro-1(+)]	18.94±5.91	35	0.133	0.0044
