# Supplementary material for: C. elegans miro-1 Mutation Reduces the Amount of Mitochondria and Extends Life Span
Source: PLoS One. 2016 Apr 11;11(4):e0153233. doi: 10.1371/journal.pone.0153233 (PMC4827821; doi:10.1371/journal.pone.0153233)
Supplement: S2 Table — (PDF) [file pone.0153233.s003.pdf]

Table S2: Life span of *daf-16* and *daf-2* double mutants.

Experiment	Genotype	Life span* (mean ± S.D.)	n	p value vs. wild type	p value vs. miro-1(-)
1	wild type	13.24± 4.7	41		
	miro-1 (tm1966)	19.34± 7.65	35	<0.001	
	daf-2	26.66± 10.62	38	<0.001	0.002
	daf-16	13.92± 3.54	38	0.47	<0.001
	miro-1;daf-2	24.43± 15.28	45	<0.001	0.116
	miro-1;daf-16	13.1± 3.33	40	0.874	0
2	wild type	17.18±3.69	38		
	miro-1 (tm1966)	22.44±6.51	32	<0.001	
	daf-2	23.65±14.99	33	0.025	0.682
	daf-16	11.97±3.31	36	<0.001	<0.001
	miro-1;daf-2	20.78±6.37	32	0.007	0.307
	miro-1;daf-16	11.68±4.43	34	<0.001	<0.001
3	wild type	15.93±5.61	41		
	miro-1 (tm1966)	23.92±8.19	37	<0.001	
	daf-2	39.97±16.06	33	<0.001	<0.001
	daf-16	14.42±3.65	36	0.1722	<0.001
	miro-1;daf-2	40.26±6.10	39	<0.001	<0.001
	miro-1;daf-16	15.26±4.63	35	0.576	<0.001

*These experiments were terminated after the last *miro-1* mutant had died, and the life span was calculated assuming the remaining worms had died immediately after. The life spans for long lived *daf-2* and *miro-1; daf-2* double mutants are possible minimal values.
