# Supplementary material for: C. elegans miro-1 Mutation Reduces the Amount of Mitochondria and Extends Life Span
Source: PLoS One. 2016 Apr 11;11(4):e0153233. doi: 10.1371/journal.pone.0153233 (PMC4827821; doi:10.1371/journal.pone.0153233)
Supplement: S3 Table — (PDF) [file pone.0153233.s004.pdf]

Table S3: Life span of *aex-6* and *rab-3* .

Experiment	Genotype	Life span (mean ± S.D.)	n	p value* vs. wild type	p value* vs. miro-1(-)
1	wild type	15.37±5.22	43		
	miro-1 (tm1966)	27.16±8.07	44	<0.0001	
	rab-3	16.53±4.93	47	0.2813	<0.0001
	aex-6	23.36±9.79	39	<0.0001	0.0562
2	wild type	15.93±5.51	41		
	miro-1 (tm1966)	23.92±8.19	37	<0.0001	
	rab-3	17.1±4.42	42	0.2885	<0.0001
	aex-6	18.71±8.45	42	0.0804	0.0069
