# Supplementary material for: C. elegans miro-1 Mutation Reduces the Amount of Mitochondria and Extends Life Span
Source: PLoS One. 2016 Apr 11;11(4):e0153233. doi: 10.1371/journal.pone.0153233 (PMC4827821; doi:10.1371/journal.pone.0153233)
Supplement: S4 Table — (PDF) [file pone.0153233.s005.pdf]

Table S4: Life span of worms treated with *ubl-5* and *atfs-1* RNAi.

Experiment	Genotype	Life span (mean ± S.D.)	n	p value* vs. control
1	*N2 ;L4440 (RNAi)*	16.48±5.45	40	
	N2 ;ubl-5 (RNAi)	14.82±5.28	49	0.1495
	miro-1;L4440(RNAi)	20.16±4.64	50	
	miro-1 ;ubl-5 (RNAi)	18.94±4.83	48	0.2053
	isp-1 ;L4440 (RNAi)	21.25±7.98	8	
	isp-1;ubl-5 (RNAi)	12.6±4.14	10	0.0089
2	*N2 ;L4440 (RNAi)*	15.32±4.64	44	
	N2 ;ubl-5 (RNAi)	14.95±5.47	42	0.7356
	miro-1;L4440(RNAi)	21.83±5.40	42	
	miro-1 ;ubl-5 (RNAi)	19.83±3.91	36	0.0689
	isp-1 ;L4440 (RNAi)	20.83±8.02	12	
	isp-1;ubl-5 (RNAi)	15.13±4.94	15	0.032
3	*N2 ;L4440 (RNAi)*	17.2±5.13	41	
	N2 ;ubl-5 (RNAi)	17.14±3.76	35	0.9545
	miro-1;L4440(RNAi)	22.23±3.96	47	
	miro-1 ;ubl-5 (RNAi)	22.0±3.33	38	0.776
	isp-1 ;L4440 (RNAi)	26.89±3.44	9	
	isp-1;ubl-5 (RNAi)	19.57±5.19	7	0.0044
4	*N2 ;L4440 (RNAi)*	14.55±3.41	31	
	N2 ;ubl-5 (RNAi)	16.67±3.05	24	0.0203
	N2;atfs-1 (RNAi)	15.57±3.53	28	0.2639
	miro-1;L4440(RNAi)	22.16±4.49	31	
	miro-1 ;ubl-5 (RNAi)	19.79±3.77	29	0.0313
	miro-1;atfs-1 (RNAi)	19.62±3.24	26	0.0195
	isp-1 ;L4440 (RNAi)	16.35±4.04	3	
	isp-1;ubl-5 (RNAi)	22±1.41	2	0.166
	isp-1;atfs-1 (RNAi)	20.44±4.3	9	0.1794
5	*N2 ;L4440 (RNAi)*	18.65±4.89	43	
	N2 ;ubl-5 (RNAi)	17.5±4.15	48	0.2283
	N2;atfs-1 (RNAi)	18.2±4.41	50	0.6419
	miro-1;L4440(RNAi)	24.4±4.78	30	
	miro-1 ;ubl-5 (RNAi)	23.35±6.03	23	0.4825
	miro-1;atfs-1 (RNAi)	21.37±5.1	30	0.0209
	isp-1 ;L4440 (RNAi)	19.38±4.90	8	
	isp-1;ubl-5 (RNAi)	23.64±5.2	11	0.0888
	isp-1;atfs-1 (RNAi)	18.67±6.8	12	0.8026
